# Vascular and neurosensory evaluation in relation to lingual canal anatomy after mandibular midline implant installation in edentulous patients

**DOI:** 10.1007/s00784-021-04312-w

**Published:** 2022-01-05

**Authors:** Mohamed Sad Chaar, Amr Ahmed Naguib, Ahmed Mohamed Abd Alsamad, Dina Fahim Ahmed, Nouran Abdel Nabi, Matthias Kern

**Affiliations:** 1 Department of Prosthodontics, Propaedeutic and Dental Materials, School of Dentistry, Christian-Albrechts University, Kiel, Germany; 2 Department of Removable Prosthodontics, Faculty of Dentistry, Cairo University, Cairo, Egypt; 3 Department of Oral and Maxillofacial Radiology, Faculty of Dentistry, Cairo University, Cairo, Egypt

**Keywords:** Mandibular midline implant, Lingual canal, Edentulous, Vascular, Neurosensory

## Abstract

**Objectives:**

The aim of this study is to investigate vascular and neurosensory complications in edentulous patients following the installation of mandibular midline single implants in relation to lingual canals.

**Materials and methods:**

After performing a cone beam computed tomography scan for the 50 recruited patients, the relationship between the potential implant site and the lingual canals was assessed, and all vascular and neurosensory complications were recorded.

**Results:**

Six patients (12%) reported profuse bleeding during implant placement, and 13 (26%) reported transient neurosensory changes, which were resolved after 3 months. According to the virtual implant planning, 44 patients (88%) would have their implants touching the lingual canals, six of them reported vascular changes (14%), and 12 out of 44 patients reported neurosensory changes (27%). For the six patients who would have their implants not touching the lingual canals, one patient reported transient neurosensory changes.

**Conclusions:**

The mandibular lingual canals are constant anatomic landmarks. Injury to the supra-spinosum lingual canals may occur during midline implant placement, depending on the implant length and the bone height.

**Clinical relevance:**

Despite that injury to the supra-spinosum lingual canals during implant insertion does not result in permanent vascular or neurosensory complications, caution is required to avoid the perforation of the lingual cortices.

## Introduction

Two dental implants in the edentulous mandible have become a standard treatment option for retaining mandibular complete dentures [1]. Installing a single implant in the midline of an edentulous mandible to retain an overdenture was introduced to make the treatment more cost-effective and less invasive [2]. Promising medium- to long-term survival rates have been reported with this treatment option [2–6].

The interforaminal region has been the most commonly used area for implant installation. However, this region contains important anatomic structures, as well as important arterial anastomoses, including multiple accessories of the lingual canals (LCs) [7, 8]. Nevertheless, the presence of the LCs has not been clearly described in dental anatomy textbooks [9], despite it has been reported even in ancient mandibles [10]. The terminology for LCs has included median lingual canal, lingual vascular canal, and mandibular genial spinal canal [11]. Moreover, they have been classified by their location in the mandible into the median lingual foramen (MLF), which is located in the midline and the lateral lingual foramen (LLF), which is laterally positioned. The associated canals of each foramen are termed as the median lingual canal and the lateral lingual canal. The median vascular canal is named according to its relationship with the genial tubercles, that is, the superior spinosum or supra-spinosum, above the genial tubercles and the inferior spinosum or infra-spinosum below the genial tubercles [12].

The supra-spinosum LC of the median lingual foramen has vascular and nerve supply that are branches of the lingual artery, sublingual artery, lingual vein, and lingual nerve [4, 9, 13, 14]. The infra-spinosum LC of the median lingual foramen has a branch of the submental or sublingual artery [13] and a branch of the mylohyoid nerve [14].

Severe hemorrhage accompanied by hematoma of the floor of the mouth has been reported during implant placement in the anterior region of the mandible [15–18] as a result of the rupture of the lingual periosteum and perforation of the lingual cortex [19]. To avoid such complications, the LCs of the mandible have been studied using cadaveric studies, panoramic radiographs, multi-slice computed tomography (MSCT), cone beam computed tomography (CBCT), and ultrasound/Doppler. Panoramic radiographs have been reported to show the LC only in 6.1% of the patients [19]. MSCT will eliminate anatomic superimposition and will give better visualization of the soft tissue, but the radiographic dose of MSCT is up to ten times higher than that of CBCT (MSCT: 430–1160 µSv and CBCT: 27–674 µSv) [20–22]. CBCT is more convenient to use and provides higher resolution on all three axes compared with the spiral CT because of the use of a 0.3-mm isotropic voxel or less [23, 24].

The submental and the lingual arteries are considered to be the two main sources of arterial blood supply to the anterior mandible. Occasionally, the arterial supply may be accompanied by nerves [25], which could lead to postoperative complications following implant installations, including anesthesia, paresthesia, or dysesthesia, depending on the degree of injury to the nerve [26]. Various methods have been used to detect such sensory disturbances, either subjectively by using questionnaires [27–29], objectively by performing physical tests [27], or by both [26]. The physical tests most often used to detect sensory changes in the anterior mandible are the two-point discrimination, the pain perception, and the temperature sensitivity tests [27].

Several studies reported major complications and life-threatening hemorrhages that occur during implant installation, but very few addressed the vascular and neurosensory changes that occur during implant installation in proximity to the LC at the midline of the mandible. Therefore, the main objective of this prospective clinical study was to investigate whether vascular complications and/or neurosensory impairment would occur following the installation of implants in the midline of edentulous mandibles in relation to the anatomy and proximity of the LCs.

## Materials and methods

The ethical committee of the Faculty of Dentistry, Cairo University, Egypt approved the study on June, 2016 (Ethical Approval Number: 16/6/10). The current study was prepared in accordance with the SPIRIT statement for reporting clinical trials [30], and performed according to the Good Clinical Practice (GCP) and to the principles of the Declaration of Helsinki (2008). Moreover, the study was registered at the PAN AFRICAN CLINICAL TRIALS REGISTRY (Trial number: PACTR20183003085193).

Eighty completely edentulous participants, seeking to improve the retention of their mandibular complete dentures by installing a single implant in the midline of the mandible, were recruited by following strict inclusion/exclusion criteria (Table 1). All participants gave informed consent prior to inclusion in the study. The 50 participants included in the current study were the first included 50 patients to receive an implant, meaning they were the first enrolled in a larger randomized clinical trial, which finally included 80 participants. These 50 participants had to sign an additional informed consent form to approve the use of neurosensory tests after implant installation. Their medical history was recorded, and a simple test was performed to assess whether there was any neuro-sensory deficiency. A cotton roll was rubbed along the right and left sides of the patient’s mandibular ridge, and the participant was asked whether they felt any difference in sensation between the right and left sides, or whether they noticed any kind of sensory disturbance. Participants were included in the randomized clinical trial according to the healing protocol and later to the attachment system. Participant age ranged from 50 to 69 years (38 men and 12 women), with a mean age of 60.9 years for men and 58 years for women. The participants’ complete sufficient dentures were duplicated to fabricate transparent radiographic stents, with radio-opaque acrylic resin placed in the anterior central incisor area. The radiographic stent was checked inside the patient mouth to be stable.

**Table 1:** Inclusion and exclusion criteria of the randomized clinical trial

Inclusion criteria:
- Completely edentulous patients, age 50–69 years
- Patients seeking to install a single median implant in the mandible to improve the stability and retention of their mandibular denture
- No contraindications for implantation
- Each participant had to undergo both a random blood sugar and glycosylated hemoglobin analysis. Participants had to have a glycosylated hemoglobin test HbA1c up to 8% [31] and a normal blood sugar level (79 to 110) or controlled diabetes (90–130 fasting according to American Association of Diabetes)
- Sufficient bone width (≥ 5 mm) in the anterior region to place an implant. It could be either normally present or achieved by bone plateauing and confirmed by CBCT scans
- The minimal residual bone height should be 11 mm and 13 mm at the posterior and the anterior regions of the mandible, respectively (Class II or III according to McGarry) [32]
- All participants should have adapted to their dentures for at least six weeks before being included in the clinical trial
- All participants should provide a written consent to participate in the trial before the scheduled date for implant installation
Exclusion criteria:
- A minimum insertion torque of 30 Ncm and/or a minimum implant stability quotient of 60 ISQ (Ostell instrument) were not achieved
- An allergic reaction to titanium
- Failure of the participant to comply with trial requirement
- Withdrawal of consent

All participants were then referred to the Oral and Maxillofacial Radiology Department for CBCT examination while wearing their radiographic stent (ProMax 3D Mid, Planmeca, Helsinki, Finland) using the standard patient positioning protocol. All participants were scanned using a tube potential of 90 kVp, a tube current of 10 mA, a cylindrical field of view of 20 × 6 cm, and a voxel size of 400 µm. The acquired images were processed and measured (Planmeca Romexis Viewer 3.5.1 software, Planmeca). All observations and measurements were carried out independently by two oral and maxillofacial radiologists experienced in CBCT scan interpretation in separate sessions.

**Fig. 1:**
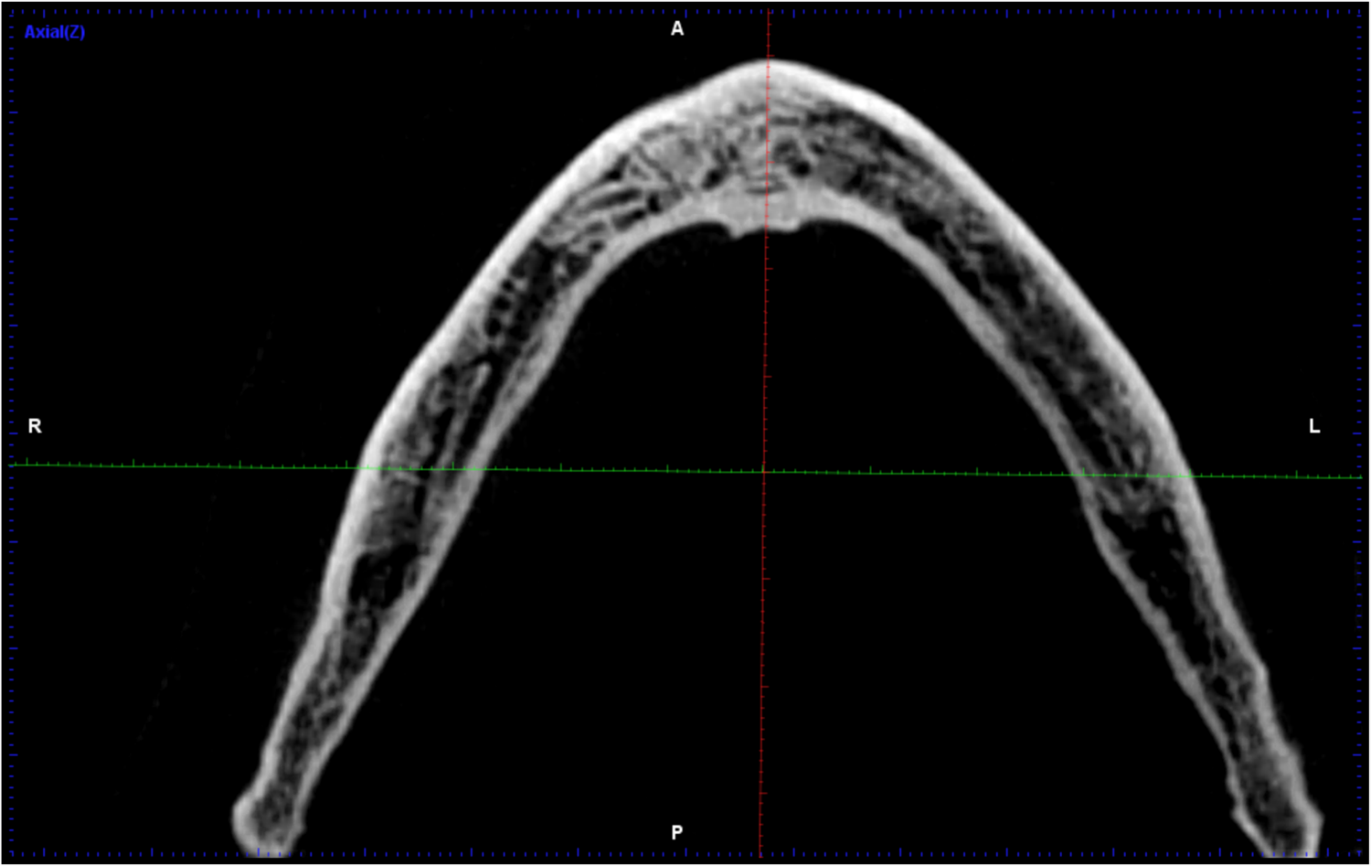
Detection of the midline from the convexity of the genial tubercle (mental spine) and mental ridge on an axial cut (slice thickness 0.4 mm and slice gap 0.4 mm)

**Fig. 2:**
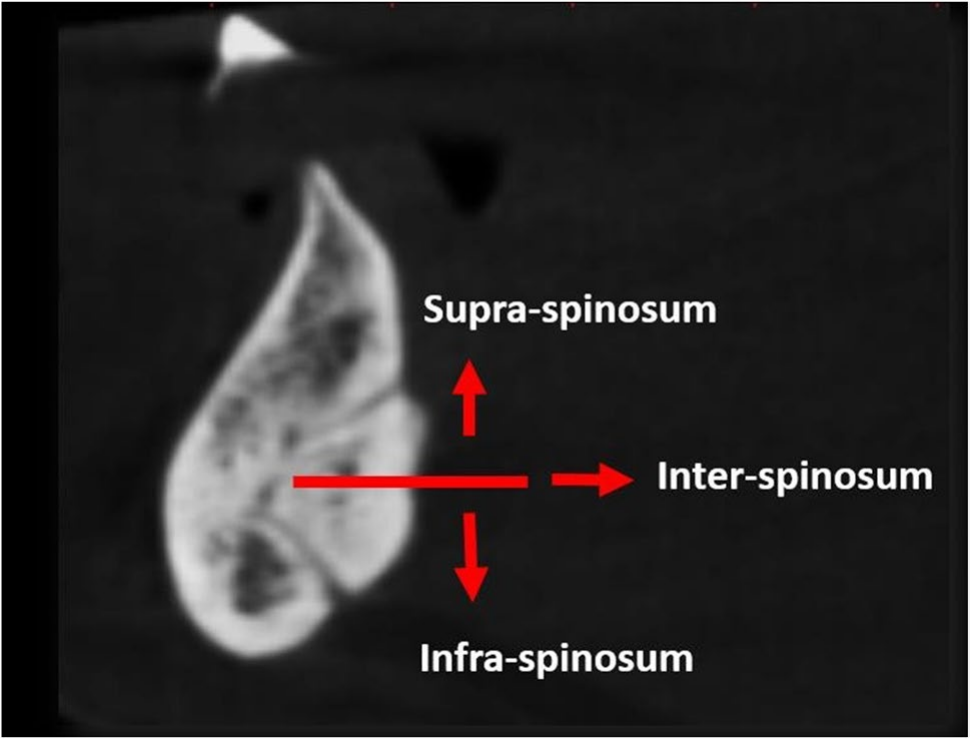
The presence, distribution, and number of canals

**Fig. 3:**
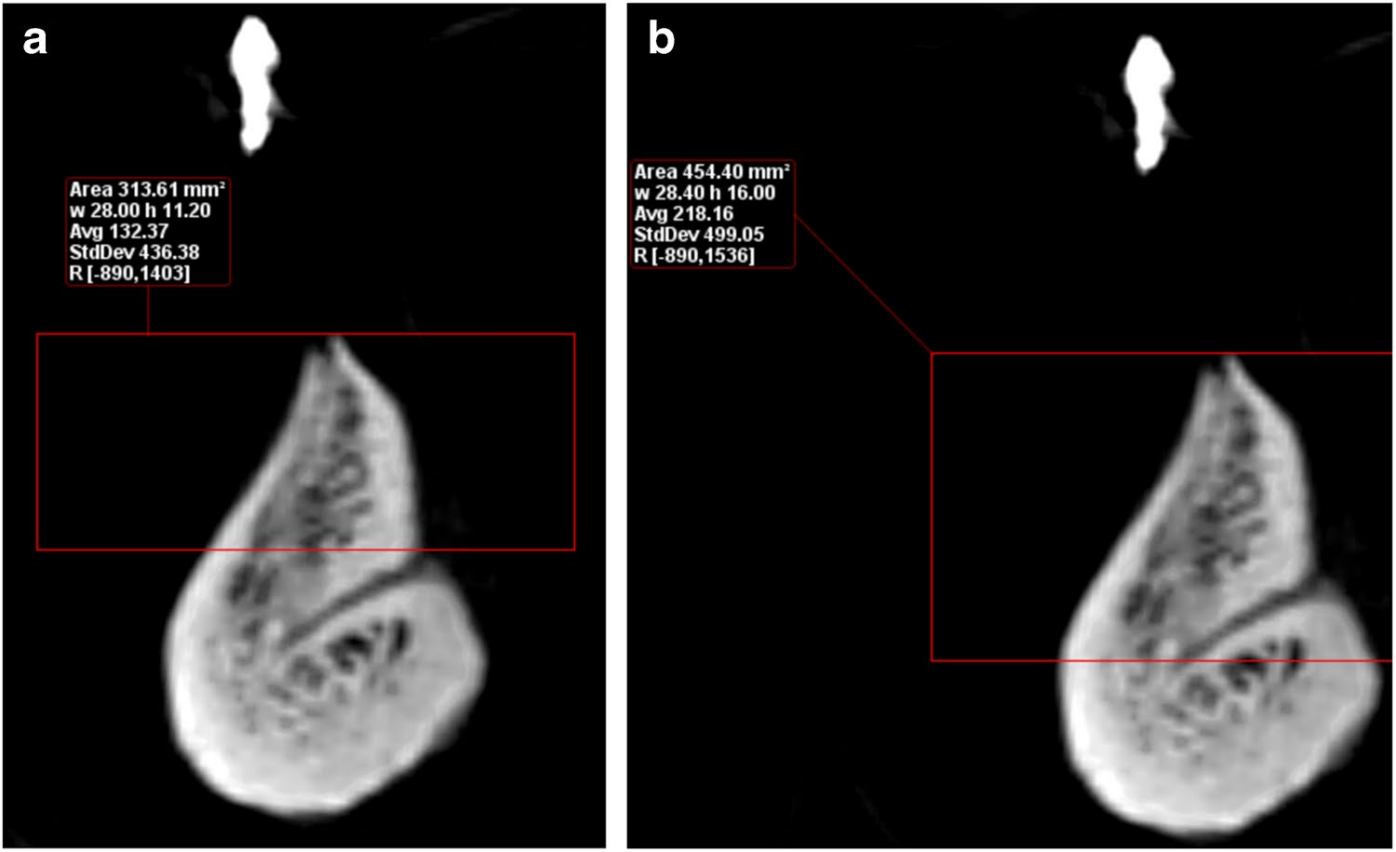
**a** Distance between the upper border. **b** Distance between the upper border of the lingual terminal end of the canal of the buccal terminal end of the canal and the alveolar crest and the alveolar crest

**Fig. 4:**
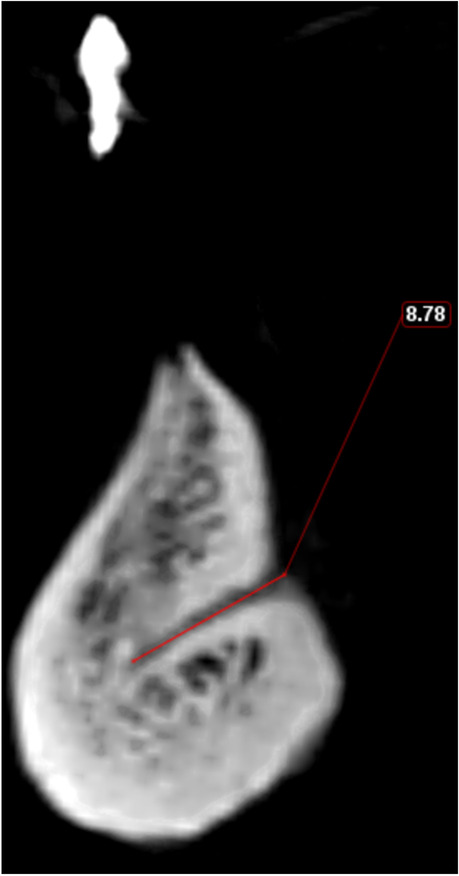
Measurement of the canal length

**Fig. 5:**
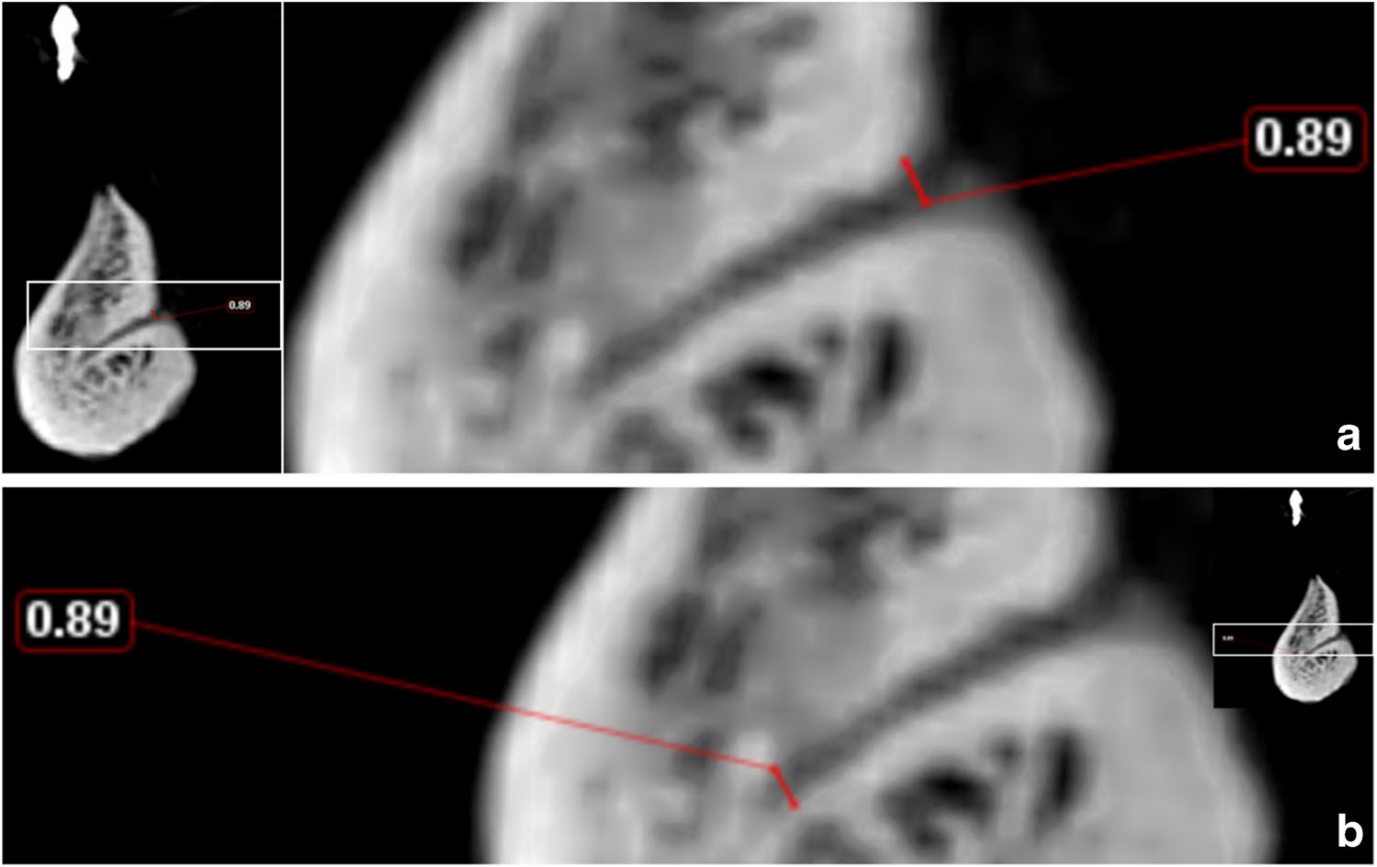
**a** The diameter of the buccal terminal end. **b** The diameter of the lingual terminal end

In order to detect the LCs, the midline was defined on the axial image by a line running between the pogonion and the apex of the mental spine of each mandible [18]. Thereafter, it was adjusted from the convexity of the genial tubercle (mental spine) and mental ridge on the axial cut using a slice thickness and slice gap of 0.4 mm each (Fig. 1), so that five consecutive sagittal cuts were assessed on each side of the midline to investigate the following aspects:

1. The LCs were classified according to their relation to the mental spine and were divided into supra-spinosum and infra-spinosum depending upon the level of opening of the canal above or below the genial spines. Supra-spinosum canals include the foramina at the level of the genial spines (Fig. 2) [33]. The morphological variations of the lingual canals in the current study were classified according to Ali and Ahmad [34]
2. The distance between the upper borders of both the buccal and the lingual terminal ends of each canal and the alveolar crest (Fig. 3a, b)
3. The length of each canal (Fig. 4)
4. The diameter of both the lingual and the buccal terminal ends of each canal (Fig. 5a, b)

The assessment of the appearance and the morphological variations of the LCs were agreed upon by the two investigators, while an average of the two readings of both investigators was reported for the linear measurements.

**Fig. 6:**
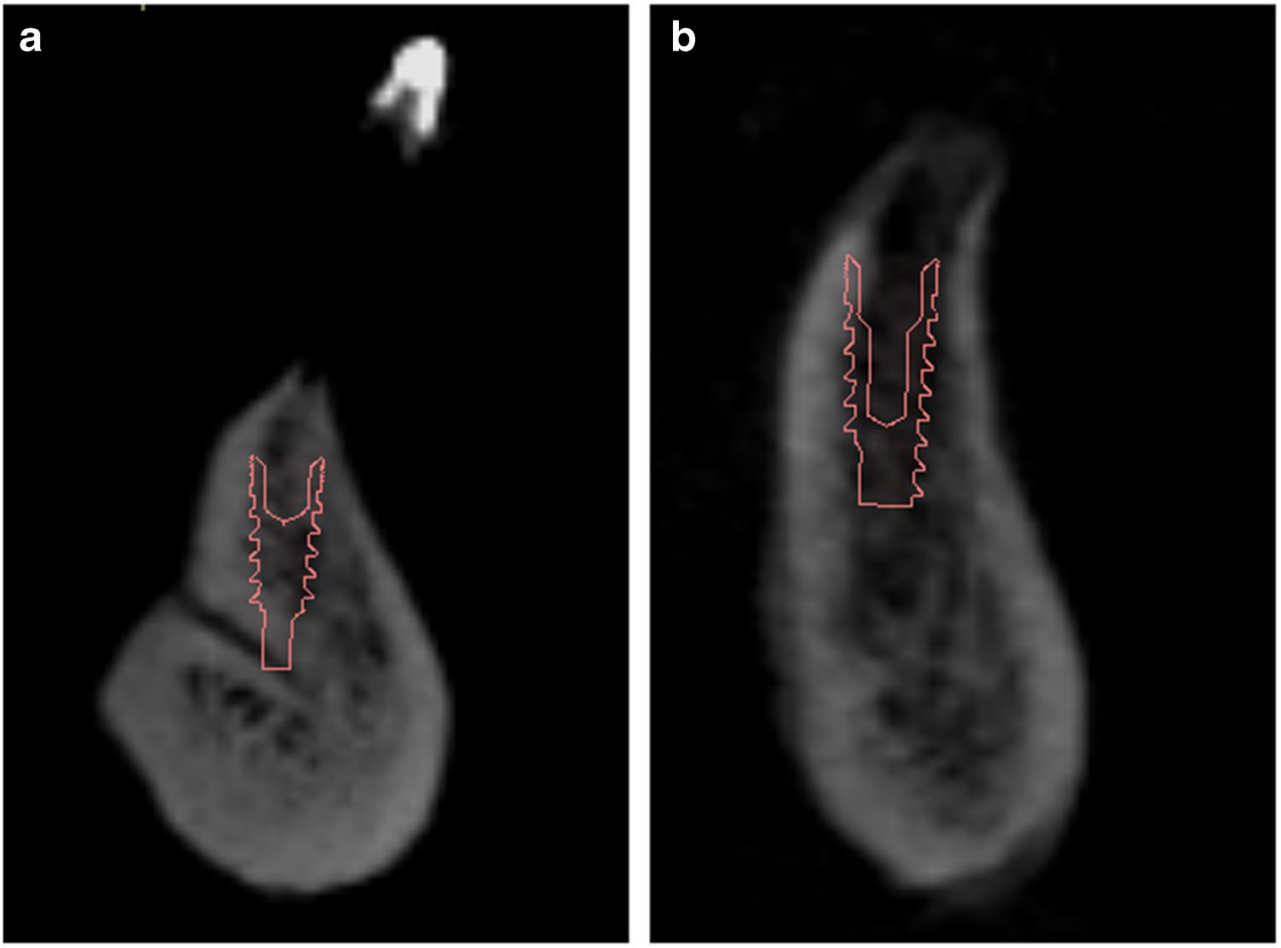
**a** Implant touching the canal. **b** Implant not touching the canal

For virtual implant planning, the same Blue Sky Bio software was used. All implants were virtually placed, guided by the radio-opaque acrylic resin positioned in the midline of the mandible (Fig. 6a, b). The relationship between the potential implant site and the LC was assessed. According to the proximity between the potential implant site and the canals, the authors proposed the following classification:

1. Touching (≤ 2 mm) (Fig. 6a), with possible injury of the LC
2. Not touching (> 2 mm) (Fig. 6b), with no possible injury of the LC

All patients were instructed to take 2000 mg of Amoxicillin as a single dose 2 h before surgery [35]. Local infiltration anesthesia was given at the mandibular anterior area, and a crestal incision (20 mm) was then made at the area of the implant installation, guided by the radiographic stent, which was converted into a surgical stent. A special attention was paid to preserve an adequate width of keratinized attached mucosa around the installed implants, particularly for non-submerged implants, which would help to have better soft and hard tissue stability, less plaque accumulation, limited soft tissue recession, and lower incidence of peri-implant mucositis and periimplantitis [36, 37].

All implants were 3.7 mm in diameter and 10 mm in length (Tapered screw vent, Zimmer Dental GmbH, Warsaw, Indiana, USA). The implant stability quotient (ISQ) was measured for all implants (Osstell, Integration Diagnostics Ltd., Sävedalen, Sweden) using the Osstell corresponding abutment (Type 32 used for Zimmer implant, Osstell), which should give a reading of at least 60 ISQ in all four directions (right, left, lingual, buccal) to be able to randomize the participant for the two healing protocols (submerged and non-submerged). In the case of lower ISQ values, the participant was excluded from the study. During implant installation, care was taken not to perforate the bone cortex and any vascular disturbance was recorded. A score (0) was given as (absent) to the absence of arterial injury (normal bleeding). While if profuse pulsating bleeding was detected and stopped (applying gauze pressure for 5 min), a score (1) was given as (present).

On the day of implant installation, the participant’s mandibular denture was relieved to avoid pressure on the underlying tissues and relined with a resilient denture liner (COE-SOFT, GC AMERICA INC, IL, USA). After 10 to 14 days from implant installation, all participants were recalled for suture removal provided that the incision site had healed properly with no signs of pain or swelling. Any vascular or sensory complications were recorded. On the day of suture removal, the following physical tests were carried out and recorded in the participant’s neurosensory chart:

1. Pin pressure: An explorer was used to exert pin pressure intra-orally at the area of implant installation and extra-orally around the lips and chin to detect the presence of sensation.
2. Static touch detection test: With the participant’s eyes closed, a cotton tip applicator was used to determine sensation in the area of the implant installation inside the mouth and extra-orally around the lips and chin.
3. Direction of movement test: With the participant’s eyes closed, a soft brush was used to determine the ability to detect both sensation and direction of movement. The soft brush was moved over the buccal and lingual mucosa at the area of implant installation and extra-orally over the lips and chin.
4. Two-point discrimination test: With the participant’s eyes closed, calipers were used to determine the ability to discriminate the varying distance between the two points of the calipers, which were about 6 mm apart. The calipers were placed over the chin, and the participant was asked whether it was felt as one point or two points.

The results of these tests were recorded, so that a score of (0) was given if there was no change detected in all the sensory tests (absent). If any change was detected, a score of (1) was given, denoting the presence of neurosensory changes.

All participants were then recalled 3 months after implant installation for the pickup stage (prosthetic loading), and the neurosensory changes were reassessed.

The detected vascular and neurosensory complications for all participants were then separately correlated with all of the following variables: gender, age, number of lingual canals, anatomic lingual foramen morphology, distance between the upper border of the buccal terminal and alveolar crest (mm), distance between the upper border of the lingual terminal and alveolar crest (mm), canal length (mm), diameter of the lingual terminal (mm), diameter of the buccal terminal (mm), as well as proximity of the virtually planned implant to the LCs in order to evaluate whether any of the variables correlated with the vascular and neurosensory changes encountered.

Data management and statistical analysis were performed using the Statistical Package for Social Sciences (IBM SPSS for Windows, Version 21.0, IBM SPSS, Inc., Chicago, IL, USA). Data were explored for normality using the Kolmogorov–Smirnov test and Shapiro–Wilk test. A two-way analysis of variance with repeated measure was used accordingly. Categorical data were summarized as percentages; differences were analyzed with χ^2^ (chi square) tests and the Fisher exact test when appropriate. Adjustments of *p* value were done using the Bonferroni method for multiple testing. To compare vascular and neurosensory complications, the Mann–Whitney was used.

## Results

No drop-out and no implant failure were reported within the healing period of three months of the 50 participants of the current study.

### Intra-class correlation of the measurements

The distance between the upper border of the buccal terminal and alveolar crest (mm), the distance between the upper border of the lingual terminal and alveolar crest (mm), the canal length (mm), the diameter of the lingual terminal (mm), and the diameter of the buccal terminal (mm) were determined by the two radiologists. They revealed a strong agreement (greater than 0.7) as shown in Table 2. Regarding a possible nerve injury, the two radiologists discussed whether the planned implant would touch or would not touch the canal to reach a consensus.

**Table 2:** Intra-class correlation **(**ICC) of the two measurements carried out by the two radiologists

Measurements	Lingual canals	ICC	95% Confidence interval	
			Lower bound	Upper bound
Distance upper border of the buccal terminal and alveolar crest (mm)	Supra-spinosum	0.767	0.59	0.867
	Infra-spinosum	0.872	0.776	0.927
Distance upper border of the lingual terminal and alveolar crest (mm)	Supra-spinosum	0.852	0.741	0.916
	Infra-spinosum	0.887	0.802	0.936
Canal length (mm)	Supra-spinosum	0.864	0.728	0.928
	Infra-spinosum	0.835	0.71	0.906
Diameter of lingual terminal (mm)	Supra-spinosum	0.760	0.568	0.866
	Infra-spinosum	0.705	0.484	0.832
Diameter of buccal terminal (mm)	Supra-spinosum	0.768	0.593	0.868
	Infra-spinosum	0.754	0.567	0.86

### Anatomic morphology of LCs

**Fig. 7:**
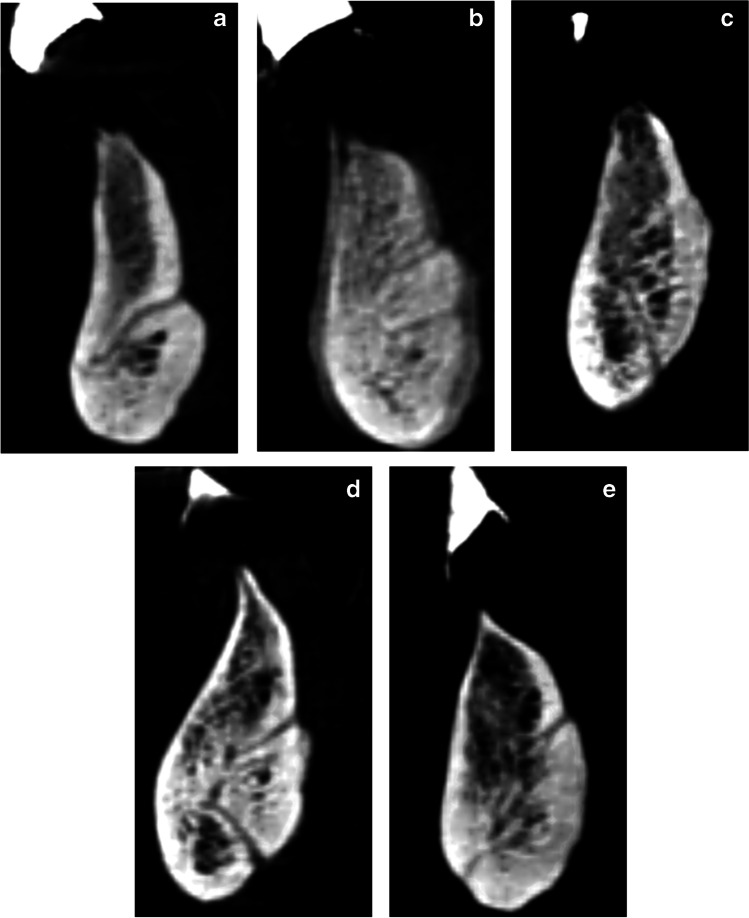
**a**–**e** Variety of canal morphology recorded in the present study. **a** Type A = one supra-spinosum canal in which the lingual terminal is higher than the buccal terminal. b Type B = One supra-spinosum canal in which the lingual terminal is higher than the buccal terminal and one infra-spinosum canal. **c** Type D = One supra-spinosum canal in which the lingual terminal is higher than the buccal terminal and one infra-spinosum canal that runs horizontally. Both are connected to each other. **d** Type E = One supra-spinosum canal in which the lingual terminal is higher than the buccal terminal and one infra-spinosum canal in which the lingual terminal is at a lower level than the buccal terminal. Both are connected to each other. **e** Other = that does not suit all of the above type

The anatomic morphology of the LCs was classified [34]. The canal morphology was of type A for 7 participants, type B for 3 participants, type D for 7 participants, type E for 8 participants, and of other type for 15 participants (Fig. 7a–e). The lingual canal classification was used in order to view the number of supra-spinosum and infra-spinosum canals with their buccal and lingual terminals.

### Number and measurements of the LCs

All participants showed at least one canal. A supra-spinosum canal was found in 49 participants, eight of them showed two supra-spinosum canals. The mean value of the supra-spinosum canals number was (1.1 ± 0.4). An infra-spinosum canal was found in 38 participants, ten of them showed two infra-spinosum canals (Table 3). The mean value of infra-spinosum canals number was (1 ± 0.7).

**Table 3:** Number of supra-spinosum, infra-spinosum canals, and lingual foramina

Lingual canals	Total number of patients with canal	Number of patients with one lingual foramen	Number of patients with two lingual foramina
Supra-spinosum canal	49	41	8
Infra-spinosum canal	38	28	10

### Linear measurements of the LCs

All the linear measurements of the lingual canals are summarized in Table 4.

**Table 4:** Means, standard deviations, and ranges of the different linear measurements of LCs

Linear measurements		Mean ± SD	Range
Distance between the upper border of the buccal terminal and alveolar crest	Supra-spinosum	14.4 ± 4.2 mm	3.6–21.2 mm
	Infra-spinosum	16.5 ± 4.4 mm	9.4–24.8 mm
Distance between the upper border of the lingual terminal and alveolar crest	Supra-spinosum	9.6 ± 3.8 mm	2.7–19.4 mm
	Infra-spinosum	19.9 ± 4.8 mm	10.8–30.4 mm
Canal length	Supra-spinosum	8.8 ± 2.1 mm	3.5–15.3 mm
	Infra-spinosum	7.1 ± 1.7 mm	2.4–10.2 mm
Diameter of lingual terminal	Supra-spinosum	0.6 ± 0.2 mm	0.4–1.1 mm
	Infra-spinosum	0.6 ± 0.2 mm	0.4–1.2 mm
Diameter of buccal terminal	Supra-spinosum	0.6 ± 0.2 mm	0.4–1.1 mm
	Infra-spinosum	0.5 ± 0.2 mm	0.4–1 mm

### Correlation of different variables with vascular complications

Only 6 participants (4 men and 2 women) experienced vascular complications during implant installation, one of them experienced sensory complications. When correlating the effect of age and gender with the vascular complications, no statistically significant effect was found (Table 5). When analyzing the morphology and the anatomy of the LCs, neither the number of canals nor the distance to the anatomic outlines had a statistically significant effect on the vascular complications reported in the present clinical study (*p* > 0.05) (Table 6). The data of both the vascular complications and sensory changes, which are presented as median and range, were not normally distributed. Thus, all *p* values were two-sided, so that a *p* ≤ 0.05 was considered statistically significant.

**Table 5:** Effect of age and gender on the vascular complications

		Vascular complications				*p* value
		Absent		Present		
		No	%	No	%	
Age (yrs.)	Mean ± SD	60.4 ± 5.0		60.2 ± 5.5		0.089
Gender	Female	10	83.3	2	16.7	0.621
	Male	34	89.5	4	10.5

**Table 6:** Effect of the anatomy and the location of the LCs on the reported vascular complications and neurosensory changes

		Vascular complications (VS)		*p* value	Neurosensory changes (NS)		*p* value
		Absent (*n* = 44)	Present (*n* = 6)		Absent (*n* = 37)	Present (*n* = 13)	
		Median (range)	Median (range)		Median (range)	Median (range)	
Number of canals	Supra-spinosum	1 (1–2)	1 (1–2)	0.435	1 (0–2)	1 (0–2)	1
	Infra-spinosum	1 (1–2)	1.5 (1–2)	0.262	1 (0–2)	1 (0–2)	0.925
Distance between the upper border of the buccal terminal and alveolar crest (mm)	Supra-spinosum	15 (3.6–21.2)	14.5 (11.2–18.8)	1	15.2 (0–20.8)	14.2 (0–21.2)	0.821
	Infra-spinosum	16.8 (9.4–24.8)	16.9 (16.4–18.2)	0.781	16.4 (0–24.8)	12.4 (0–22.4)	0.166
Distance between the upper border of the lingual terminal and alveolar crest (mm)	Supra-spinosum	9.2 (2.7–16.8)	9.3 (7.8–10.0)	0.925	9.4 (0–17)	8.6 (0–15)	0.634
	Infra-spinosum	19.4 (10.8–30.4)	20.6 (16.8–21.4)	0.982	19.4 (0–30)	15.4 (0–28)	0.142
Canal length (mm)	Supra-spinosum	8.8 (3.5–15.3)	9.3 (6.4–12.3)	0.585	9 (0–15.3)	8 (0–11.2)	0.127
	Infra-spinosum	7.1 (2.4–10.6)	7.7 (4.3–8.0)	0.871	6.5 (0–10.2)	6.9 (0–10.6)	0.617
Diameter of lingual terminal (mm)	Supra-spinosum	0.5 (0.4–1.1)	0.6 (0.5–1)	0.269	0.5 (0–1.1)	0.5 (0–0.9)	0.814
	Infra-spinosum	0.5 (0.4–1.2)	0.5 (0.4–1)	0.887	0.5 (0–1)	0.6 (0–1.2)	0.076
Diameter of buccal terminal (mm)	Supra-spinosum	0.5 (0.4–1.1)	0.6 (0.5–0.9)	0.144	0.5 (0–1.1)	0.5 (0–1)	0.941
	Infra-spinosum	0.5 (0.4–1.0)	0.5 (0.4–0.7)	0.844	0.4 (0–1)	0.4 (0–1)	0.312

When correlating the proximity of the virtually placed implant to the LCs, 44 participants had their implants within 2 mm to the canal (with possible injury), and 6 participants had their implants away from the canal (with no possible injury). Even so, only 6 participants showed vascular complications in the group who had their planned implants in close proximity to the canal. In the group who had their planned implant away from the canal, no vascular complications were reported. Being in close proximity to the LC (possible injury of the canal) showed no statistically significant correlation with the reported vascular complications (*p* > 0.05) (Table 6).

#### Correlation of the different variables to neurosensory changes

Overall, 13 participants (26%; 6 women and 7 men) out of all 50 participants reported transient neurosensory changes following implant placement, all of which disappeared within 3 months. Only one participant experienced both vascular complications and sensory changes. Age did not show significant effect on the neurosensory complications. However, concerning the gender of the participants, the neurosensory complications were statistically significantly higher in females (50%) than in males (18.6%) (*p* = 0.03) (Table 7).

**Table 7:** Effect of age and gender on the neurosensory complications

		Neurosensory Complications				*p* value
		Absent		Present		
		No	%	No	%	
Age(yrs.)	Mean ± SD	60.6 ± 5.3		59.5 ± 3.9		0.508
Gender	Female	6	50.0	6	50.0	0.030
	Male	31	81.6	7	18.4	

Likewise, the number of canals, the proximity to the anatomic outlines, the canal length, the diameter of the lingual terminal, and the diameter of the buccal terminal showed no statistically significant effect on the reported neurosensory changes (Table 6).

Forty-four participants had their virtually placed implants touching the lingual canal, 12 of whom experienced neurosensory changes and 32 of whom did not. Of the 6 participants who had their implants virtually away from the canal (not touching), only one experienced neurosensory change. The virtual proximity of the planned implants to the lingual canal showed no statistically significant correlation with the neurosensory changes (*p* > 0.05) (Table 8).

**Table 8:** Proximity of the virtually placed implant to the LCs and vascular complications and neurosensory changes

Proximity to the LCs	Vascular complications				*p* value	Neurosensory complications				*p* value
	Absent		Present			Absent		Present		
	No	%	No	%		No	%	No	%	
No	6	100	0	0	0.335	5	83.3	1	16.7	1
Yes	38	86.4	6	13.6		32	72.7	13	27.3	

## Discussion

Implant installation in the midline of the mandible may be very challenging due to the presence of lingual canals and their associated blood vessels and nerve innervation [9, 11]. Several studies reported complications such as life-threatening hemorrhages and other neurosensory complications [15–17, 26]. Therefore, the main aim of this clinical trial was to try to understand the correlation between the reported vascular and neurosensory complications and the proximity to the lingual canals and their anatomy, which of high clinical relevance to guide clinicians during implant planning and placement.

In order to report the vascular complications and neurosensory changes related to implant installation in proximity to the LCs of the midline of a completely edentulous mandible, different factors were considered, including age, gender, location/anatomy of the LCs, and proximity of the virtually planned implants to the canals.

The LCs were detected using CBCT scans as they have been reported to be 4–20 times more accurate than panoramic radiographs [21, 38]. One study concluded that CBCT is more accurate in detecting the various maxillary and mandibular anatomic structures than panoramic x-rays [36]. Moreover, to overcome variation in the quality of the image due to slice thickness, two oral and maxillofacial radiologists independently detected and measured all of the LCs following a defined protocol to give more consistent evaluation [39]. Moreover, standardized settings were used for all CBCT scans.

During the osteotomy and implant installation, only 6 of 50 participants (12%) exhibited profuse bleeding, which was easily arrested by applying gauze pressure for 5 min; no hemorrhagic complications were reported postoperatively. Overall, 13 (26%) of the participants experienced transient neurosensory changes after implant installation, which disappeared within 3 months. Both the vascular and neurosensory complications showed a non-statistically significant correlation with the considered factors (patient and LC related).

According to the results of this study and given that 88% of the installed implants were probably touching the lingual canal (based on the virtual planning), no serious vascular or permanent neurosensory complications were encountered. One explanation is that all installed implants were 10 mm in length, which is considered an optimum implant length in the anterior inter-foraminal region [7]. The life-threatening hemorrhages reported in the literature mostly occurred with an implant length and osteotomy preparation of greater than 15 mm [7]. Moreover, most of them occurred because the lingual cortical plate was perforated [40], resulting in hematoma and swelling in the floor of the mouth and leading in turn to obstruction of the upper airway space [12, 22, 24, 41–43]. An interesting review of literature concluded that during implant installation in the mandible, a proper planning is mandatory to avoid perforation of the lingual cortex otherwise severe bleeding could occur, which would require specialized care [44]. Furthermore, one study reported after macro-anatomic dissection of 20 mandibles, the close proximity of blood vessels to the lingual cortical plate of the midline of the mandibular, which would increase the risk of bleeding even with minimal perforation of the mandibular lingual plate [9]. Therefore, during implant installation in the mandible, proper planning is mandatory to avoid the perforation of the lingual cortex and its associated severe bleeding. No hemorrhages were reported in the current study, which is in agreement with other studies that reported hemorrhages following implant installation in a completely edentulous mandible but in other regions, i.e., two patients hemorrhaged in the first premolar region, seven in the canine region, and only one in the region of the lateral incisor, indicating the least number of hemorrhages in the lateral incisor area [43–50]. Another explanation could be that in elderly and edentulous patients, the central blood supply could be compromised [51, 52].

The neurosensory change was described as temporary, according to the classification of Seddon and Sunderland and may be classified as “Neurapraxia” and defined as temporary when the interruption of nerve transmission is caused by nerve compression, edema, hematoma, and minor stretching. Furthermore, the recovery rate is fast, ranging from days to 12 weeks [53, 54]. However, keeping a safe distance of 2 mm between implants and the neurovascular bundle of the lingual foramen is still recommended to avoid these transient vascular/neurosensory complications [55].

In edentulous patients, the need to reduce the vertical bone height because of limited crestal bone thickness might result in close proximity to the LCs during implant installation. The average distance calculated in the present study of the supra-spinosum median LC to the crest of the ridge was 9.6 ± 3.8 mm, which means that the supra-spinosum LC should always be taken into consideration during midline implant planning and placement. The average distance of the infra-spinosum median lingual canal to the crest of the ridge was 19.9 ± 4.8 mm, which appears to be a safe distance, and is in agreement with other studies [12, 24, 42, 56–59] that have reported an average safe distance of 15.5 mm from the median lingual foramen to the crest.

The diameter of the median lingual foramen can also put the patient at higher risk of hemorrhage if it is greater than 1 mm, but only if the lingual cortex is perforated [9]. The average diameter of the supra-spinosum median lingual canal and that of the infra-spinosum median lingual canal in the present study was 0.6 ± 0.2 mm and 0.5 ± 0.2, respectively, which is considered to be within the safe range. A systematic review concluded that the diameter of the LC is an important anatomic variation to be considered during implant installation in the midline of the mandible [60]. A similar study using CBCT scans was carried out on edentulous cadaveric mandibles to detect possible contact between virtually placed single midline implants and the lingual canals and concluded that the risk of midline implants contacting the lingual canal is high [61]. Likewise, another macro- and microanatomical study concluded after the dissection of 12 intact cadaver mandibles that the superior and inferior genial spinal foramina have different neurovascular contents, determined by their anatomical location above or below the genial spines [13].

They recommended to carry out further clinical studies to report the possible complications of midline implant installation and their clinical relevance. A clinical trial using CBCT scans investigated the importance of detecting the presence, position, and size of the lingual foramina before implant installation in dentate patients and reported that the lingual foramina were present below and above the tooth apex, that those above the tooth apex were of smaller diameter, and that the distance between them and the tooth apex changed with increasing age [58].

Moreover, a recent anatomical study curried on dentate and edentulous cadaveric mandibles investigated the genial spinal canal histologically concluded that the genial spinal canal in the dentate specimen contained a neurovascular bundle, which branched into a nerve innervating the incisor and a neurovascular bundle, whereas that in the edentulous specimen contained some nerves for vestibular gingival innervation and a vascular bundle [62]. The results propose differences in the genial spinal canal between dentate and edentulous mandibles and may explain the low incidence of both vascular or neurosensory complications in the current study. Hence, our clinical study was carried out to report the complications of midline implant installation in the completely edentulous mandible in relation to lingual canals, which is of greater clinical relevance than the detection itself.

A limitation of the current study was the lack of a postoperative CBCT scans to evaluate the actual proximity and possible injury of placed implants to the LCs and their relationship to the reported vascular and neurosensory complications. A second CBCT scan was not possible as it was not clinically justified and therefore not approved by the ethics committee. Only a standardized peri-apical radiograph was approved to evaluate the bone height changes.

## Conclusions

From the present study, it can be concluded that the mandibular lingual canals are constant anatomic landmarks that are present at the midline of the mandible, as all participants showed at least one canal and 98% of them had a supra-spinosum canal (49 of 50 participants). There is considerable potential for injury to the supra-spinosum lingual canals during midline implant placement, depending on the implant length and the available bone height. Injuries of the supra-spinosum lingual canals during implant insertion in the midline of the mandible without perforation of bone cortices did not result in permanent vascular or neurosensory complications.
